# Supplementary material for: Estimating excess mortality during the COVID-19 pandemic from a population-based infectious disease surveillance in two diverse populations in Kenya, March 2020-December 2021
Source: PLOS Glob Public Health. 2023 Aug 23;3(8):e0002141. doi: 10.1371/journal.pgph.0002141 (PMC10446178; doi:10.1371/journal.pgph.0002141)
Supplement: S1 Table — NCD-Non-Communicable Diseases; CoD-Cause of Death. (DOCX) [file pgph.0002141.s001.docx]

**S1 Table:** Proportion of causes of death pre-and-during COVID-19 Period in Kibera, Kenya, 2016-2021

| COD | 2016 | 2017 | 2018 | 2019 | 2020 | 2021 |
| --- | --- | --- | --- | --- | --- | --- |
| *Accidental drowning and submersion* | - | - | 2.1 | - | 4.7 | - |
| *Accidental exposure to smoke fire & flame* | - | - | 2.1 | - | - | - |
| *Accidental fall* | - | - | - | - | - | 3.5 |
| *Accidental poisoning & noxious substance* | - | - | - | 2.3 | - | - |
| *Acute abdomen* | 5.1 | 2.9 | - | - | 2.3 | - |
| *Acute cardiac disease* | - | - | 4.3 | - | 2.3 | 5.3 |
| *Acute respiratory infection incl. Pneumonia* | 17.9 | 21.4 | 6.4 | 2.3 | 11.6 | 1.8 |
| *Assault* | 5.1 | 1.4 | 4.3 | 2.3 | 2.3 | - |
| *Asthma* | 7.7 | 2.9 | - | - | - | - |
| *Birth asphyxia* | 2.6 | - | 4.3 | 2.3 | 4.7 | 5.3 |
| *Breast neoplasms* | - | - | - | - | - | 1.8 |
| *Congenital malformation* | - | - | - | 2.3 | 2.3 | - |
| *Diabetes mellitus* | - | - | 2.1 | - | - | 1.8 |
| *Diarrhoeal diseases* | - | - | 4.3 | - | - | 5.3 |
| *Digestive neoplasms* | 7.7 | - | 4.3 | 2.3 | 4.7 | 1.8 |
| *Ectopic pregnancy* | - | 1.4 | - | - | - | - |
| *Epilepsy* | - | - | 2.1 | 7.0 | 2.3 | 3.5 |
| *Exposure to force of nature* | - | - | 2.1 | - | - | - |
| *Haemorrhagic fever* | 2.6 | - | - | - | - | - |
| *HIV/AIDS related death* | 12.8 | 20.0 | 12.8 | 30.2 | 18.6 | 15.8 |
| *Indeterminate* | 5.1 | 2.9 | - | - | 2.3 | 3.5 |
| *Intentional self-harm* | - | 1.4 | - | - | - | - |
| *Liver cirrhosis* | - | - | - | 7.0 | - | 1.8 |
| *Malaria* | 5.1 | 5.7 | 2.1 | - | - | - |
| *Meningitis and encephalitis* | 2.6 | 1.4 | 2.1 | 7.0 | 2.3 | 3.5 |
| *Neonatal pneumonia* | - | 2.9 | - | - | - | 1.8 |
| *Obstetric haemorrhage* | - | - | - | - | 2.3 | - |
| *Other and unspecified cardiac disease* | 2.6 | 2.9 | 6.4 | 4.7 | 2.3 | 3.5 |
| *Other and unspecified external CoD* | - | 1.4 | 2.1 | 2.3 | 2.3 | - |
| *Other and unspecified infectious disease* | - | 4.3 | - | 7.0 | - | 5.3 |
| *Other and unspecified NCD* | - | - | 2.1 | - | - | - |
| *Other and unspecified neoplasms* | 2.6 | - | - | - | 2.3 | 3.5 |
| *Pregnancy-induced hypertension* | - | - | - | 2.3 | 2.3 | 1.8 |
| *Prematurity* | - | 2.9 | 4.3 | 2.3 | 7.0 | 5.3 |
| *Pulmonary tuberculosis* | 5.1 | 8.6 | 8.5 | 4.7 | 4.7 | 1.8 |
| *Renal failure* | 2.6 | 2.9 | - | - | - | 1.8 |
| *Reproductive neoplasms mf* | - | 1.4 | - | 2.3 | 2.3 | 3.5 |
| *Respiratory neoplasms* | - | 1.4 | - | - | - | 5.3 |
| *Road traffic accident* | 10.3 | 7.1 | 4.3 | 4.7 | 11.6 | 7.0 |
| *Severe malnutrition* | - | 1.4 | 2.1 | - | - | 1.8 |
| *Sickle cell with crisis* | - | - | 2.1 | 2.3 | - | - |
| *Stroke* | 2.6 | 1.4 | 12.8 | 2.3 | - | 3.5 |
| *Tetanus* | - | - | - | - | 2.3 | - |

NCD-Non-Communicable Diseases; CoD-Cause of Death
