# Supplementary material for: Estimating excess mortality during the COVID-19 pandemic from a population-based infectious disease surveillance in two diverse populations in Kenya, March 2020-December 2021
Source: PLOS Glob Public Health. 2023 Aug 23;3(8):e0002141. doi: 10.1371/journal.pgph.0002141 (PMC10446178; doi:10.1371/journal.pgph.0002141)
Supplement: S2 Table — NCD-Non-Communicable Diseases; CoD-Cause of Death. (DOCX) [file pgph.0002141.s002.docx]

**S2 Table:** Proportion of causes of death pre- and during COVID-19 Period in Asembo, Kenya,2016-2021

|  | **2016** | **2017** | **2018** | **2019** | **2020** | **2021** |
| --- | --- | --- | --- | --- | --- | --- |
| *Accidental drowning and submersion* | 0.8 | 1.3 | - | - | - | - |
| *Accidental exposure to smoke fire &flame* | - | - | 0.9 | - | - | - |
| *Accidental fall* | - | 0.9 | - | - | - | - |
| *Acute abdomen* | 3.1 | 3 | 3.1 | 1.9 | 4 | 1.1 |
| *Acute cardiac disease* | 1.9 | 1.3 | 0.4 | 1 | 1.8 | 2.8 |
| *Acute respiratory infection incl. Pneumonia* | 14.3 | 14.7 | 16.2 | 13.6 | 14.7 | 21.6 |
| *Anemia of pregnancy* | - | 0.4 | - | - | 0.4 | - |
| *Assault* | 1.9 | 2.2 | 1.3 | 1 | 1.8 | 2.8 |
| *Asthma* | 1.2 | 1.3 | 2.6 | 2.9 | - | 1.7 |
| *Birth asphyxia* | 0.4 | 0.9 | 2.6 | 2.9 | 1.3 | 1.7 |
| *Breast neoplasms* | - | 0.4 | 0.4 | - | 0.4 | 0.6 |
| *Chronic obstructive pulmonary disease* | 1.9 | 1.7 | 1.7 | 1.9 | 1.3 | 1.7 |
| *Congenital malformation* | 0.8 | 0.9 | - | 1.5 | 0.4 | - |
| *Contact with venomous plant/animal* | 0.4 | - | - | - | - | - |
| *Diabetes mellitus* | 2.3 | 5.2 | 0.9 | 1.9 | 2.2 | 3.4 |
| *Diarrhoeal diseases* | 0.8 | 0.4 | 0.4 | 0.5 | 0.4 | - |
| *Digestive neoplasms* | 1.6 | 0.9 | 0.9 | 1 | 0.4 | 2.8 |
| *Epilepsy* | 0.8 | 1.3 | 1.7 | 0.5 | 1.8 | 1.1 |
| *Hemorrhagic fever* | - | - | - | 0.5 | - | - |
| *HIV/AIDS related death* | 17.4 | 15.2 | 8.7 | 9.2 | 10.3 | 9.1 |
| *Indeterminate* | 3.9 | 3 | 3.5 | 3.4 | 2.2 | 1.1 |
| *Intentional self-harm* | 0.4 | - | - | - | 0.4 | 0.6 |
| *Liver cirrhosis* | 0.4 | - | 1.3 | 0.5 | - | - |
| *Malaria* | 12.8 | 4.8 | 6.1 | 9.2 | 7.6 | 11.4 |
| *Meningitis and encephalitis* | 0.4 | 0.4 | - | - | 0.9 | - |
| *Neonatal pneumonia* | 0.8 | 3.5 | 1.7 | 1.9 | 1.3 | 0.6 |
| *Neonatal sepsis* | - | 0.4 | 1.3 | 1.5 | 0.9 | - |
| *Oral neoplasms* | - | - | 0.4 | - | - | - |
| *Other and unspecified cardiac disease* | 7.8 | 7.8 | 15.3 | 14.6 | 14.3 | 14.8 |
| *Other and unspecified external CoD* | 0.4 | 1.3 | - | - | - | - |
| *Other and unspecified infect disease* | 0.8 | 0.9 | 0.9 | 0.5 | 0.9 | - |
| *Other and unspecified maternal CoD* | - | - | 0.4 | - | - | - |
| *Other and unspecified NCD* | 0.4 | - | 0.4 | 1 | 0.9 | - |
| *Other and unspecified neonatal CoD* | 0.8 | 0.9 | - | 0.5 | 0.4 | - |
| *Other and unspecified neoplasms* | 1.2 | 1.7 | 2.2 | 1.9 | 2.2 | 1.7 |
| *Other transport accident* | - | - | 1.3 | 1 | 0.4 | 1.1 |
| *Pertussis* | - | - | 0.4 | - | - | - |
| *Pregnancy-induced hypertension* | - | 1.3 | - | 0.5 | - | - |
| *Pregnancy-related sepsis* | **-** | 0.4 | **-** | **-** | **-** | **-** |
| *Prematurity* | 0.4 | 1.3 | 0.4 | - | 0.9 | - |
| *Pulmonary tuberculosis* | 8.1 | 9.1 | 9.2 | 5.8 | 5.4 | 1.7 |
| *Renal failure* | 1.9 | 0.4 | 0.9 | 1 | 1.8 | 0.6 |
| *Reproductive neoplasms mf* | 0.8 | 0.9 | 0.4 | 0.5 | 1.8 | 1.1 |
| *Respiratory neoplasms* | 2.3 | 3.9 | 4.4 | 2.9 | 1.8 | 1.1 |
| *Road traffic accident* | 0.8 | 0.9 | 2.2 | 3.4 | 2.7 | 2.8 |
| *Sepsis (non-obstetric)* | - | 0.4 | - | - | 0.4 | - |
| *Severe anaemia* | 1.2 | 0.9 | 3.9 | 2.9 | 4.5 | 6.3 |
| *Severe malnutrition* | 1.2 | 0.9 | - | 0.5 | 1.3 | 1.1 |
| *Sickle cell with crisis* | 0.4 | - | - | 0.5 | - | - |
| *Stroke* | 3.5 | 3 | 1.3 | 5.8 | 5.4 | 3.4 |

NCD-Non-Communicable Diseases; CoD-Cause of Death
