# Supplementary material for: Estimating excess mortality during the COVID-19 pandemic from a population-based infectious disease surveillance in two diverse populations in Kenya, March 2020-December 2021
Source: PLOS Glob Public Health. 2023 Aug 23;3(8):e0002141. doi: 10.1371/journal.pgph.0002141 (PMC10446178; doi:10.1371/journal.pgph.0002141)
Supplement: Attachment — Submitted filename: Response to Reviewers.docx [file pgph.0002141.s004.docx]

June 15, 2023

To Academic Editor,
PLOS Global Public Health

Dear Humayun Kabir

**Ref.: PGPH-D-23-00661: Estimating excess mortality during the COVID-19 pandemic from a population-based infectious disease surveillance in two diverse populations in Kenya, March 2020-December 2021**
 On behalf of my co-authors, I wish to thank you for inviting us to submit a revised draft of our manuscript. We also appreciate the time and effort you and all the reviewers have dedicated to providing very insightful comments. Therefore, we resubmit the edited manuscript for further consideration.

Again, thank you for giving us the opportunity to strengthen our manuscript.

Sincerely,

Clifford Oduor,

Corresponding author

**Response to Reviewers’ Comments**

*(All responses are highlighted in blue)*

**Reviewer #1**

Results Section:
*Do we know what percent of the all cause mortality rates are attributed to COVID-19? For example - for people who died of respiratory illnesses, do we know what percent of these deaths are a side effect of COVID-19 related illnesses?

Response: Thanks for raising this question. We noted that the highest change in proportion of deaths during COVID-19 period in both Kibera and Asembo site were as a result of ARI/pneumonia. However, we were not able to ascertain specifically what percentage of the all-cause mortality were attributed to COVID-19 or were COVID-19 related. We acknowledge this as a limitation and have included this in line 248-251.page 10

* How does the increase in vaccination rates reflect on the mortality rates? Do we anticipate a reduction in the mortality rates once the vaccination rates arise?

Response: Increase in COVID-19 vaccination coverage would definitely reduce the COVID-19 related mortality. In this study we were not able to assess the impact of COVID-19 vaccination on mortality rates. However, we hypothesize that the lack of significant change in mortality rates in our urban site could have been attributed to a relatively high COVID-19 vaccination coverage in urban areas compared to rural areas in Kenya. We have added this point in the discussion in line 235-236, page 10

**Reviewer #2**

The Manuscript is scientifically sound. easy for a leader to follow through and understand the processes in carrying out the study. The research was able to provide all the statistical methods used to analyze the data even the methods of data collection which is in line with the type of study carried out. The manuscript was written in a manner that it provides enough data to justify carrying out the research, the outcome and the limitation of the study were elaborated which is important for providing insights of the gaps that the study had. I recommend this research for publishing. However, try to explain further on the differences in mortality of the whole data as at first sight without the analysis you might conclude that the period March 2020 to 2021 had increase in deaths for the two study locations compared to the period 2016 to February 2020. if you put into considerations the time periods. The first time period had a different of 4 years to the comparison group which is just a year. I suggest explaining more of deaths in reduced time periods rather than comparing the longer period to the shorter period.
All in all, congratulations for a job well done in providing scientific data on mortality during this time period as statistics had shown that there was not much mortality due to Covid -19 in Africa Compared to other parts of the world.
Note: data for the comments above has been highlighted in the attachment.

Response: The authors wish to thank the reviewer for the positive feedback and suggestion.

We wish to state that we generated the mortality rates by using the person years of observation(pyo) denominator which accounted for differences in population size and death counts given by the different time period in the comparison groups. The robust nature of this approach allowed us to conduct a comparison of the mortality rates between the groups. This approach is highlighted in line 93-95, page 4

**Reviewer #3**

MY dear authors many thanks for this value paper and simple writing
*Regarding the line 170 to 173 regarding specific causes of death pre COVID-19 Period by age group in Kibera and Asembo you remember the mortality reduction at both urban and rural for pulonary tuberculosis patients during covid i ask whats type of pulomnary is active or controlled ( closed case ) on DOT or not negative sputum or positive so reduction at mortality regarding these pulmonary T.B during covid may be due another factors like DOT treatment , nore health care supervision or admitted patients.

Response: The authors wish to thank the reviewer for raising this question. Unfortunately, due to the limited scope of the study we were not able to ascertain what type of pulmonary TB deaths were impacted on. We also acknowledge that it is not clear the factors that might have led to the reduction in TB deaths in this setting.
*you not remember the smoking as risk factor if has positive or negatively effect

Response: Thank you for providing this insight. We acknowledge that we were not able to assess whether smoking had any effect particularly on the deaths due to respiratory illnesses.

*the rural araea like Asembo incresed mortality may due to many factors related to education level or health facilities or tradition about covid-19 virsu or lach of mass media health information so high mortality may has many causes direct or undirect. Finally many thanks for your efforts

Response: Thank you again for providing this insight. We acknowledge that in rural areas several determinants including high poverty rates, low health literacy levels, cultural norms, educational disparities, distance to facility have been identified as barriers to healthcare access. This consequently result to high mortality rates in this setting. We have added this point in line 226-228, page 10

**Reviewer #4**

*The 95% Confidence intervals can be written as: 95% C.I example: The all-cause mortality rate was higher during the COVID-19 period compared to the pre-COVID-19 period in Asembo [9.1, 95% CI: 8.2 – 10.0) vs. 7.8, 95% CI: 7.3 – 8.3) per 1000 person-years of observation, pyo]
Response: Thanks for noting this. We have made the edit in line 17 of the abstract.
*The figure showing the causes of death could be classified into Non-communicable diseases and Communicable diseases.

Response: Thank you for this suggestion. However, we decided to give finer details in order to clearly understand the true burden of COVID-19 on specific illnesses in this population

**Reviewer #5**

This is a good paper. A few comments for the authors.
*what is the design of the study?

Response: The study was a longitudinal Population-Based Infectious Disease Surveillance. We have clarified this in the method in line 74, page 4
*What is the size of the data?

Response: In the pre-COVID-19 period, there were 285 deaths in Kibera and 1,025 deaths in Asembo. During the COVID-19 period there were 118 deaths in Kibera and 445 deaths in Asembo. We have highlighted this in line 125-127, page 5
* Did you use all the data generated in Asembo and Kibera from 2006-2021?

Response: We used data, including all registered deaths from participants of all age groups between 1st January 2016 and 31st December 2021.Data collected between 1st January 2016 to 29th February 2020 was used to generate the counterfactuals for the post- COVID-19 period (March 2020 to December 2021). This is highlighted in line 98 and line 104, page4

* Is there any reason for choosing Verbal Autopsy interviews (using next of kin, health workers and relatives) to ascertain cause of death rather than assessing the medical records of these deceased patients?

Response: Thanks for this question. We conducted the surveillance in a resource-poor-settings which often lack accurate, complete and timely reporting of in-hospital deaths. In addition, health care utilization is generally low with barriers being high cost and access. Consequently, deaths occurring outside health facilities are not well counted and causes of death remain unregistered. Therefore, faced with these challenges we opted to use population-based surveillance Verbal autopsy (VA) to assess cause-specific mortality. We have clarified this in the introduction section in line 48-50, page 3

*Please change the tenses in the methods section to the past

Response: Thanks for the suggestion. Since this is an ongoing population-based infectious disease surveillance project we opted to use present tense.
